# Association between Sexual Cognition and Sexual Dysfunction in Mexican Adult Women Survivors of Sexual Abuse

**DOI:** 10.1192/j.eurpsy.2026.12052

**Published:** 2026-07-31

**Authors:** Y. S. Ramírez Ruz, L. Carrillo Ramírez, I. Fernández Castillón

**Affiliations:** 1 Psychiatry, Tecnológico de Monterrey, School of Medicine and Health Sciences; 2 Psychiatry, Mexican Social Security Institute (IMSS), Monterrey, Mexico

## Abstract

**Introduction:**

Sexual abuse (SA) is a public health problem in Mexico, affecting 12.7% of women and associated with a high risk of female sexual dysfunction (FSD) present in 30-66% of cases. Beyond the biological, sexual cognition, defined as mental representations about sexuality, influences attitudes and sexual response. Its negative form may perpetuate dysfunction and psychological distress in cases of sexual abuse.

**Objectives:**

To determine the association between sexual cognition and sexual dysfunction in adult female survivors of sexual abuse treated in a psychiatric hospital in Nuevo León, Mexico.

**Methods:**

A cross-sectional, descriptive, and observational study was conducted between May and July 2024, with the participation of 50 women with a history of sexual abuse. Sociodemographic and clinical variables were collected, and six validated questionnaires were applied: Index of Female Sexual Function (IFSF), Arizona Sexual Experience Scale (ASEX), Female Sexual Self-Schema Scale (validated in Mexican population), PHQ-9, GAD-7, and PCL-5. Statistical analysis was done through descriptive, correlational and logistic regression models.

**Results:**

The mean age of the participants was 29.4 years. Abuse onset occurred at a mean age of 15.5 years, with an estimated duration of 79.2 weeks; 70% of the women reported having suffered multiple episodes. The prevalence of FSD was 74% according to the IFSF and 50% according to the ASEX. A significant association was observed between negative sexual cognition and sexual dysfunction (ASEX, p = 0.002), which translated into a 6-fold increased risk of presenting FSD in women with negative sexual self-schemas (OR = 6.7; 95% CI: 1.9-23.3). A relationship was also observed between negative cognition and Post-Traumatic Stress Disorder (p = 0.001), while no associations were identified with depression, anxiety, sociodemographic factors, or the type or frequency of abuse.

**Conclusions:**

A high prevalence of FSD was found in women with a history of sexual abuse, associated with negative sexual cognitions, independently of sociodemographic variables. This suggests that psychological factors, such as beliefs about sexuality, play a key role in dysfunction. This reflects the need for interventions focused on cognitive restructuring and comprehensive sexuality education to improve sexual health and quality of life in this vulnerable population.

**Disclosure of Interest:**

None Declared

